# Challenges to an effective response for addressing stigma and discrimination related to HIV: from denial of rights to construction of support networks

**DOI:** 10.7448/IAS.16.1.18931

**Published:** 2013-12-01

**Authors:** Renata Karina Reis, Marli Teresinha Gimeniz Galvão, Elucir Gir

**Affiliations:** 1 Department of General and Specialized Nursing, University of São Paulo at Ribeirão Preto College of Nursing, WHO Collaborating Centre for Nursing Research Development, Ribeirão Preto, São Paulo, Brazil; 2 Department of Nursing, Postgraduate Course in Nursing, Federal University of Ceará, Fortaleza, Brazil; 3 Department of General and Specialized Nursing, University of São Paulo at Ribeirão Preto College of Nursing, WHO Collaborating Centre for Nursing Research Development, Ribeirão Preto, São Paulo, Brazil

More than 30 years have passed since the discovery of HIV. Several important advances have been made in that time, and we can now celebrate a drop in new infection rates [1]. In addition, HIV-positive individuals have greater access to highly active antiretroviral therapy, which has promoted greater survival and a reduction in opportunistic infections [2].

Despite the important scientific advances that have been made, stigma and discrimination still create major challenges that must be overcome if we are to have a meaningful and lasting response to HIV. These challenges carry a high cost in human suffering [3] and in the violation of the human rights of people living with HIV (PLHIV) across the world [2]. They are obstacles that stand in the way of making the necessary transformations to reduce new HIV infection rates and associated diseases and deaths.

**Figure 1:**
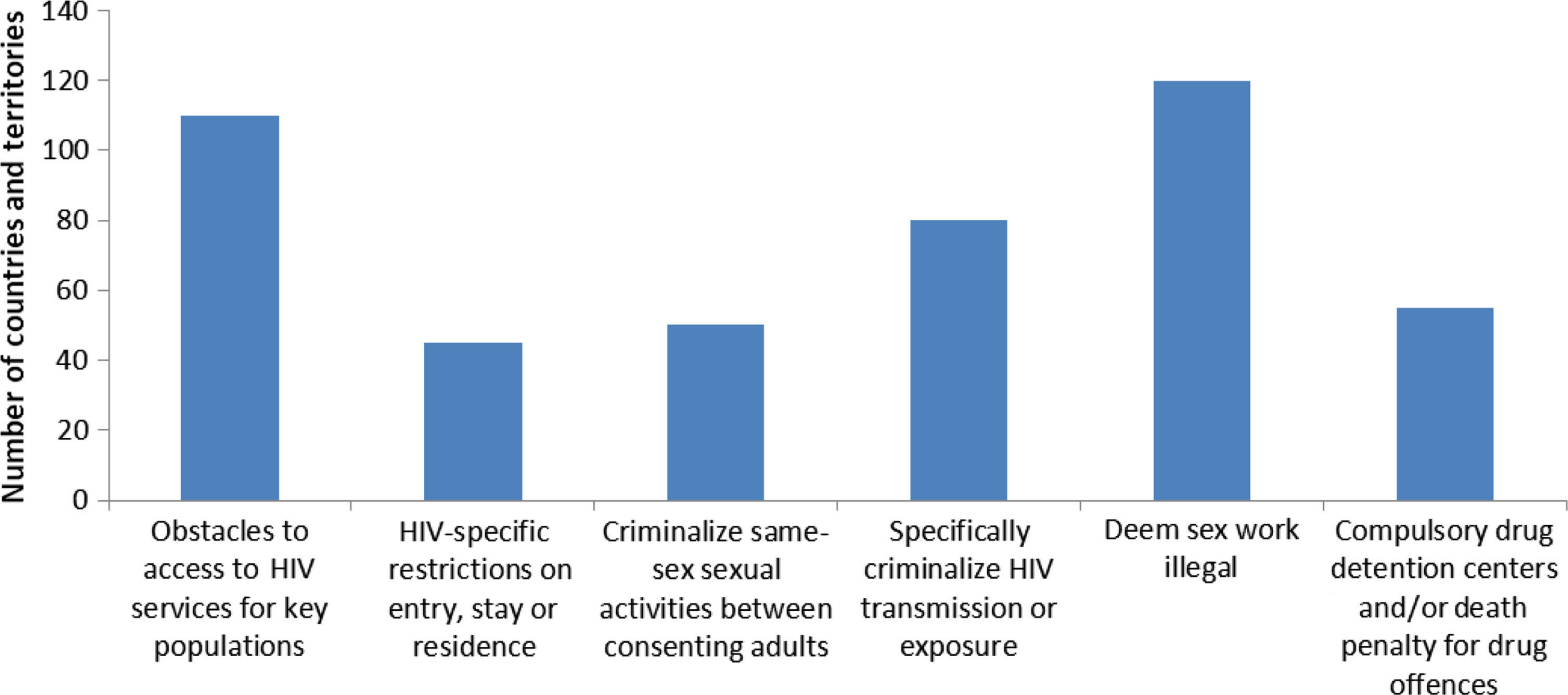
Number of countries and territories with selected laws or practices that impact on eliminate HIV related stigma and discrimination. **Source: UNAIDS 2013 (2)**

Stigma and discrimination are among the most negative and harmful social responses, and they have been present since the start of the AIDS pandemic [4]. There is even more discrimination towards PLHIV in countries with certain social, cultural and economic contexts, negatively impacting on universal access to prevention, treatment and healthcare related to HIV [5]: some countries fuel stigma and discrimination related to HIV by adopting laws and punitive practices related to transmission and exposure to HIV, sex work, use of drugs or sexuality [6] (see Figure 1).

Effective approaches to HIV-related stigma remains scantly documented in the scientific literature. Intervention studies for stigma reduction have been carried out in communities and among health professionals and students [7], and there are few reported interventions aiming at the empowerment and mobilization of PLHIV. Studies from eastern countries are particularly scarce as in India [8].

One of the main difficulties for a patient who receives a diagnosis of HIV/AIDS is disclosing it to others. Patients can face many different attitudes from their families and friends: they can be met with solidarity, support and understanding, but also with criticism, stigma, social discrimination and ruptures in relationships and life projects. These negative responses create a social death [9], which produces physical and mental suffering [10].

Afraid of suffering discrimination and the exclusion that goes with stigma, PLHIV develop attitudes of denial and they become isolated, keeping their seropositivity secret [11]. They find themselves forced to omit important aspects of their lives and facing embarrassing situations, such as lying about visiting a physician, hiding away to take the antiretrovirals, and fearing being recognized as HIV positive by someone from their social circle while visiting a health service. This affects their emotional [12], professional [13] and social lives, and also how they deal with their own treatment [14].

All of these obstacles violate the rights of PLHIV and severely interfere with how they organize their daily living, thus diminishing their chances of being happy, maintaining their health and achieving quality of life [11].

It is important to acknowledge that stigma and discrimination related to HIV/AIDS have different effects on men, women, children and adolescents [15]. Children suffer obstacles in, for example, their right to education [16] and adolescents experience obstacles in their right to leisure, privacy, anonymity and confidentiality and in their emotional life [17].

Women make up half of PLHIV, with growing numbers in many countries [2]. They are also are more susceptible to being excluded, abandoned and isolated and to suffering physical, sexual and psychological violence because of gender inequalities related to cultural standards [8]. A study conducted in India showed that members of a husband's household tend to blame the HIV-positive married woman for the disease and for the misery brought on the entire family; furthermore, the woman is blamed for not being able to keep the man from staying on the right path and is often denied shelter and the right to live in the matrimonial home, sometimes even while her HIV-positive husband is alive [8].

Across the globe, there is remarkable violation and negligence by health professionals and services [18,19] when it comes to the sexual and reproductive rights of PLHIV. This legitimates discrimination, made worse by the absence of services to adequately support family planning [20,21] and to accommodate the needs of couples living with HIV/AIDS, especially serodiscordant couples [12].

The response to coping with stigma and discrimination related to HIV is complex and demands the implementation of joint and intersectorial interventions [4]. Even when PLHIV are not summarily excluded from their social contexts, they are condemned by being labelled as incapable and unproductive. For these reasons, regaining denied citizenship means recovering social bonds, assuring compliance with the law, claiming the creation of protective instruments to the rights of PLHIV, and demanding the fulfilment of rights already guaranteed in legal instruments. Some countries, such as Fiji, Guyana, Congo, Guinea, Senegal and Togo, have begun reviewing such laws, restricting their application or dropping them altogether, as recommended by the Global Commission on HIV and the Law [2].

To guarantee human rights, gender equality and equity, important structural changes are necessary to reduce both gender inequalities and stigma towards HIV. The intensification of interventions aimed at enabling women to achieve higher levels of education and increase their participation in the workforce may promote their socio-economic independence, thus improving their empowerment and access to services of health promotion, prevention and support [15].

Another important strategy is implementation of actions to promote the right to information and to the means of prevention of HIV and awareness regarding the rights of PLHIV. This involves educational projects for the community in different social context (schools, universities, companies, churches, health services) to disseminate information regarding HIV/AIDS, rights, citizenship and violation of rights, aimed at deconstructing prejudicial and discriminatory attitudes and at awakening ethical and supportive behaviour so as to build a network of social support based on respect and solidarity. A systematic review including 14 target populations in 28 countries demonstrated effectiveness of the implementation of interventions to reduce HIV-related stigma in both industrialized and developing contexts [22].

Furthermore, it is fundamentally important to mobilize and empower PLHIV regarding their rights and citizenship so that they can both claim their rights and identify violation of their rights and react. Examples of successful experiences of interventions that contribute to the mitigation of HIV stigma are described in a study exploring networked groups of PLHIV in Uganda [23]. In Brazil, meanwhile, the call for rights of PLHIV through social mobilization resulted in laws and social policies for the regulation of control of blood banks, health plans and universal access to antiretrovirals [24].

The complexity of living with HIV, from the point of view of access to healthcare and prevention services, imposes a need for urgent changes in the health field and in healthcare models and practices: by ignoring the needs and demands that result from the reality that PLHIV now have broadened life perspectives, health professionals offer fragmented, exclusionary and reductionist care, violating the right of PLHIV to life, love, the construction of family and professional projects, health and happiness.

Some countries (e.g., Brazil, Cameroon, Cote d'Ivoire, Fiji, Madagascar, Malawi, Mauritania, Republic of Moldova and Togo) have aligned or fully integrated strategic planning and budget cycles for HIV and health generally. Available evidence suggests that integrated approaches are beneficial, enhancing service uptake and improving coordination of care. Examples include integrating tuberculosis and HIV services, services to prevent mother-to-child transmission integrated with maternal and child healthcare, linking HIV and chronic non-communicable diseases and, more broadly, HIV services integrated within primary healthcare and overall health and community systems [2].

The struggle with stigma and discrimination related to HIV/AIDS demands the development of joint and intersectorial strategies and interventions, with the mobilization of different social actors to cope with this complex issue; the construction of a support network is crucial for an effective fight against exclusion, inequality and prejudice.

There is an urgent need for more investments in public and social policies to reduce the level of social and gender inequalities [2], as well as the development of actions for implementation, monitoring and evaluation of interventions against stigma and discrimination in different social and cultural contexts so as to disseminate successful experiences that may contribute to mobilizing and empowering PLHIV/AIDS.
